# Severe Symptoms of Mental Disorders Among Students Majoring in Foreign Languages in Vietnam: A Cross-Sectional Study

**DOI:** 10.3389/fpubh.2022.855607

**Published:** 2022-05-30

**Authors:** Nguyen Thi Thang, Dao Thi Dieu Linh, Ta Nhat Anh, Nguyen Thị Phuong, Nguyen Duc Giang, Nguyen Xuan Long, Dao Thi Cam Nhung, Khuong Quynh Long

**Affiliations:** ^1^Department of Educational Psychology, University of Languages and International Studies, Vietnam National University, Hanoi, Vietnam; ^2^Center for Population Health Science, Hanoi University of Public Health, Hanoi, Vietnam

**Keywords:** social factors, foreign languages, student, Vietnam, symptoms of mental disorders

## Abstract

Mental health disorders among university students have been a serious issue in many countries and can negatively affect academic performance and all aspects of daily living, relationships, and physical health. In this study, we aim to estimate the prevalence of severe symptoms of mental disorders and examine associated factors among students majoring in foreign languages in Vietnam. We used the Depression, Anxiety, and Stress Scales (DASS-21) to detect severe symptoms of mental disorders in 1,788 students, including severe symptoms of depression, anxiety, and stress. The Financial-Study-Family-Friend (FSFF) scale was utilized to assess how much students worried about finance, academic, and social support from family and friends. Multiple logistic regressions were carried out to evaluate the relationship between severe symptoms of mental disorders and FSFF factors. The prevalence of severe levels of depression, anxiety, and stress were 21.1, 35.0, and 16.3%, respectively. While varying somewhat due to students' demographic characteristics, all four aspects of the FSFF scale were related to the severe symptoms of mental disorders of students. Concerns about study had the largest effects (ORs ranging from 2.84 to 3.72) while difficulty in finance had the smallest effects (ORs ranging from 1.23 to 1.37) on students' depression, anxiety, and stress. The prevalence of severe symptoms of mental disorders in students was high in our study. Teachers and universities should focus more attention on students' mental health. Social support from family and friends also plays a crucial role in the mental health of students.

## Introduction

Mental health is the foundation for general well-being and manifests itself in effective functioning in life. Mental health is not only the absence of mental disorders but also includes the ability to think, learn, and understand one's feelings and the reactions of others (1). Nearly one billion people globally have mental disorders (MD); severe MD tend to reduce life expectancy by 10 to 20 years in members of the general population (2). Suicide is the second leading cause of death for young people aged 15–29 and accounts for ~800,000 deaths each year (2). Unfortunately, access to quality mental health services remains insufficient in many places around the world, especially in low- and middle-income countries, where more than 75% of people suffer from MD (2).

The transition to college, which for the majority of youth coincides with the transition to emerging adulthood, is stressful and disruptive (3). Previous studies indicated that the prevalence of mental health disorders among university students is higher than that in the general population, of whom 35% suffer from depression and anxiety (4). In a study of 4,184 French students (5) the prevalence of symptoms of depression and anxiety was 12.6, and 7.6%, respectively. Another study of Chinese students showed that the prevalence of depressive symptoms was 11.7% (6).

The factors contributing to MD among students may include living away from their family and friends, entering adulthood and having to adapt to student life, financial burden, and non-self-determined motivation (3, 5, 7, 8). Other factors implicated in psychological morbidity among students include academic pressure, demanding workloads (9), concerns about personal health (10), physical inactivity (11), student abuse and mistreatment (12), reduced academic achievement, substance abuse, violence, and poor reproductive and sexual health (13). MD among students can negatively impact their academic performance (14). MD also can affect many areas of students' lives, students' lives, including motivation and concentration, which may reduce their quality of life, academic achievement, physical health, and satisfaction with the college experience, and negatively impact relationships with friends and family members. These issues can also have long-term consequences for students, affecting their future employment, earning potential, and overall health (14–16).

In Vietnam, several studies have been conducted to evaluate the prevalence of MD and related factors among undergraduate students (17). The prevalence of stress, anxiety, and depression ranges from 10.1 to 18.6% (11). However, most previous studies among language learners focused on anxiety regarding the learning process, while other MD in students, such as depression and stress, were rarely mentioned (18–20). Besides, according to the screening results of the University of Languages and International Studies (ULIS) Psychological Center, which is a center established by ULIS to help students and teachers to screen for mental health problems, foreign language students have the high prevalence of symptoms of mental disorders (SoMD). However, to the best of our knowledge, there has been limited research on symptoms of mental disorders among students of foreign languages. This article aims to examine the severe SoMD of students majoring in a foreign language at a Vietnamese university (specifically, their levels of anxiety, depression, and stress), and to determine how social factors impact their mental health, allowing specific measures to improve the quality of their mental health to be formulated and proposed.

## Methods

### Study Design and Participants

This was a cross-sectional study conducted in 2 months, from August to October 2021 at a foreign languages university in Hanoi, Vietnam.

The participants of this study were 1,788 students of the University of Foreign Languages in Hanoi, a university specializing in language education, linguistics, and international studies that educates graduates majoring in foreign languages, meeting the urgent need for qualified teachers and officials with foreign language competence. Participants included first- to fourth-year students from eight faculties: the Faculty of Japanese Language and Culture, the Faculty of Chinese Language and Culture, the Faculty of German Language and Culture, the Faculty of French Language and Culture, the Faculty of Russian Language and Culture, the Faculty of Korean Language and Culture, and the Faculty of English Language Education. Participants were recruited based on the following criteria: (1) aged 18 years old and above, (2) enrollment at the University of Foreign Languages in Hanoi, Vietnam; (3) being students majoring in foreign languages; and (4) having the physical and psychological capacity to fill the questionnaires.

### Sampling Procedure

An online survey was conducted. Students were invited to participate through an anonymous link distributed via email to students at the University of Foreign Languages and International Studies. The study was also announced by the UPC and the student communications departments. A set of screening questions was included at the beginning of the survey to ensure that students met the inclusion criteria of the study. The survey was open for 2 months, from August to October 2021. One reminder email was also sent to obtain a higher response rate. Results were reported separately according to faculty and year of study. At the time surveyed, there were approximately 5,000 full-time students at the University of Foreign Languages who were eligible to participate in the study. Most of the students were from the northern provinces of Vietnam. Of which 1,778 students accepted and submitted the completed questionnaire, accounting for 35.6%.

### Measurement

#### Dependent Variables

This study measured severe SoMD using the Depression, Anxiety, and Stress Scales (DASS-21). The DASS-21 is a widely used screening tool to assess SoMD in community settings. The DASS-21 has been validated and used in many previous studies in Vietnam, showing good reliability and validity (21, 22).

This instrument is a self-report questionnaire comprised of 21 items, with seven items per subscale, measuring three aspects: depression, anxiety, and stress. Participants rated the DASS-21 using a four-point Likert rating scale ranging from 0 “did not apply to me at all” to 3 “applied to me very much or most of the time.” Sum scores are computed by adding up the scores on the items per subscale and multiplying them by a factor of two. Sum scores for each of the subscales may range between 0 and 42, with the higher score indicating greater severity of symptoms.

The symptomatology of all three aspects (depression, anxiety, and stress) are expressed using five levels: normal, mild, moderate, severe, and extremely severe. The classifications of scores for depression levels are: 0–9 (normal), 10–13 (mild), 14–20 (moderate), 21–27 (severe), and 28+ (extremely severe); for anxiety, levels are: 0–7 (normal), 8–9 (mild), 10–14 (moderate), 15–19 (severe), and 20+ (extremely severe); for stress, levels are: 0–14, 15–18, 19–25, 26–33, and 34+, representing normal to extremely severe (23). In this study, participants who had severe to extremely severe symptoms of MD were classified as having severe SoMD.

#### Independent Variables

##### Financial-Study-Family-Friend (FSFF) Scale

To evaluate factors related to severe SoMD among students, the FSFF scale was designed and piloted.

This questionnaire was comprised of 20 items, with 6–8 items per subscale, measuring three aspects: financial burden (6 items), difficulty in learning (6 items), and social support (family: 5 items; friends: 3 items). Responses were given using a four-point Likert scale ranging from 0 (very strongly disagree) to 3 (very strongly agree). The scores for items 13 to 20 (i.e., family and friend subscales) were reversed. The mean score for each scale was then obtained, with higher values indicating students having more problems in this aspect.

#### Covariates

The covariates included the socioeconomic characteristics of the participants: age, gender, majoring in languages; academic performance (GPA); financial factors (financial resources, perceived financial situation, and tuition and living expenses); and history of stress, anxiety, or depression.

### Statistical Methods

#### Properties of FSFF

The internal consistency of the FSFF was measured using Cronbach's alpha. A Cronbach alpha coefficient of from 0.6 to 0.7, from 0.7 to 0.8, and above 0.80 indicates poor, adequate, and good internal consistency, respectively. To identify the factorial structure of FSFF, exploratory factor analysis (EFA) was applied. Before conducting EFA, the Kaiser-Meyer-Olkin test was conducted to measure the sampling adequacy for factor analysis, with values >0.7 considered to be appropriate. Parallel analysis was used to decide the number of factors retained. The principal factor extraction method and Promax oblique rotation were then applied to assume existing correlations between factors.

The correlation between FSFF and DASS-21 subscale scores was assessed using Pearson's correlation coefficient, which ranges from 0 to 1; a higher value indicates a better linear correlation.

#### Relationship Between FSFF and Severe SoMD

Multivariable logistic regressions were carried out to evaluate the relationship between FSFF subscale scores with severe SoMD. Two sets of models were fitted. In the partially adjusted models, we fitted each component of FSFF (i.e., financial, study, family, and friend) for each model separately, while in the fully adjusted model, all four aspects were included in the same model. All models were further adjusted for age, gender, monthly living expenses, GPA, and family medical history. A *p*-value of <0.05 was considered statistically significant. All analyses were conducted using Stata version 17 (Stata Corp, College Station, TX).

### Ethical Consideration

The study was approved by the Institutional Review Board of the Hanoi University of Public Health in Vietnam (IRB decision no. 325/2021/YTCC-HD3). All participants were given information about the study and notified of their rights as study participants. They also provided their informed consent voluntarily.

## Results

### Participant Characteristics

A total of 1,788 participants enrolled in the study, including 126 male and 1,662 female students. Students' demographic characteristics are shown in Table 1. More than one-third of the participants were 19 years old (40.3%) with no family history of stress, anxiety, or depression (93.1%). The percentage of students with a family history of stress, anxiety, and depression was 6.9%. Nearly half of the participants had financial support from their families (46.4%). About 74% of respondents had tuition and living expenses of about $150–195 (USD)/month and had a normal perceived financial situation (63.1%). The majority of students have a GPA ranging from 2.5 to 3.59 (83.2%).

**Table 1:** Participant characteristics.

	**Male** ***n*** **(%)**	**Female** ***n*** **(%)**	**Total** ***n*** **(%)**
	***N* = 126**	***N* = 1,662**	***N* = 1,788**
**Age**			
22	13.5	14.7	14.6
21	34.1	22.0	22.8
20	14.3	22.9	22.3
19	38.1	40.4	40.3
**Family history of stress, anxiety or depression**			
No	93.7	93.0	93.1
Yes	6.3	7.0	6.9
**Financial resources**			
Family	46.8	46.3	46.4
Part-time job	13.5	10.5	10.7
Scholarship, other	39.7	43.1	42.9
**Perceived financial situation**			
Very difficult	4.8	4.3	4.3
Difficult	15.1	16.8	16.7
Normal	57.9	63.5	63.1
Comfortable	29.0	14.1	14.5
Very comfortable	3.2	1.3	1.4
**Tuition and living expenses**			
150–195 (USD)	78.2	73.4	73.7
200–239 (USD)	8.9	13.8	13.4
240–304 (USD)	4.8	6.9	6.7
310–435 (USD)	8.1	6.0	6.1
**GPA**			
Under 2.0	0.8	1.1	1.1
2.0–2.49	8.7	6.3	6.4
2.5–3.19	34.9	43.5	42.9
3.2–3.59	44.4	40.0	40.3
3.6–4	11.1	9.2	9.3

### Prevalence of Depression, Anxiety, and Stress

**Figure 1:**
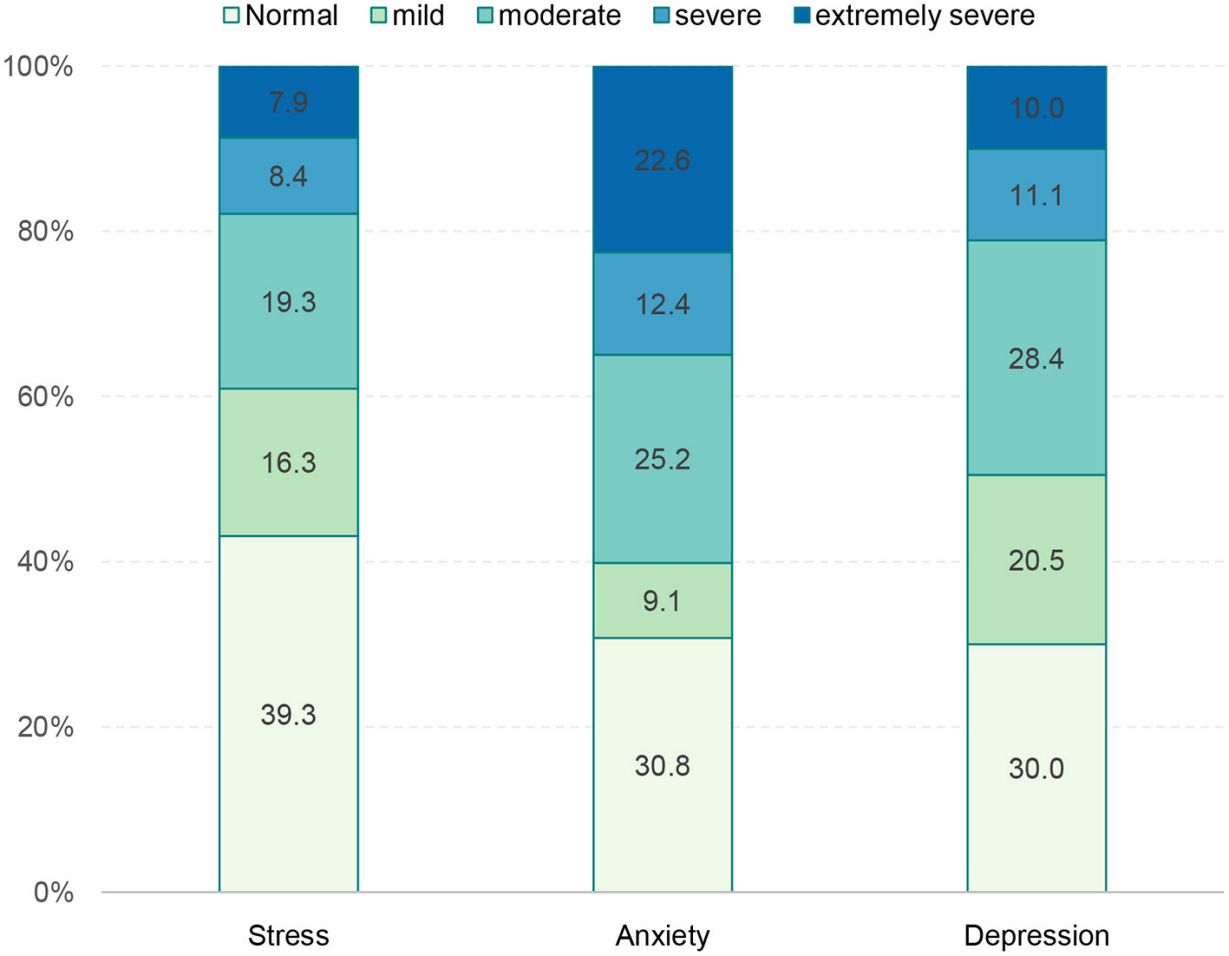
Prevalence of depression, anxiety, and stress.

Figure 1 shows the prevalence of stress, anxiety, and depression status among participants. The prevalence of symptoms of stress, anxiety, and depression among students was 83.7, 65.0, and 78.9%, respectively. A high prevalence of severe and extremely severe mental health problems was found, with 8.4 and 7.9% related to stress, 12.4 and 22.6% to anxiety, and 11.1 and 10.0% to depression, respectively.

### Properties of FSFF Scale

Table 2 presents the properties of the FSFF scale. Using EFA, 20 items from the original set of 23 items remained in the final set. Four latent factors were found to explain these 20 items: items 1 to 6 were explained by the finance factor, and items 7 to 12, items 13 to 17, and items 18 to 20 were explained by the study, family, and friend factors, respectively. The factor loadings were high in all four aspects, ranging from 0.62 to 0.83 in finance, 0.34 to 0.74 in study, 0.71 to 0.73 in family, and 0.57 to 0.80 in friends.

**Table 2:** Properties of FSFF scale.

	**Factor loading**			
	**Finance**	**Study**	**Family**	**Friend**
I'm worried about the cost of tuition	0.71			
I've calculated daily living expenses	0.61			
I worry about finding a part-time job every month	0.61			
My family's financial support is not enough for me to live and study	0.68			
I hope to receive financial support from sources outside my family	0.62			
I worry about tuition and daily living expenses	0.83			
I wonder about my future career		0.34		
I have difficulty completing academic tasks		0.74		
I try to find a way to study that works for me personally		0.60		
I am overloaded with academic deadlines		0.63		
I'm obsessed with personal academic achievement		0.62		
I need help with my studies		0.69		
I can talk about my problems with my family^*^			0.71	
I received support and encouragement from my family^*^			0.85	
I feel comfortable talking to my family members^*^			0.84	
My family supports me in making decisions^*^			0.76	
My family really tries to help me^*^			0.73	
I can talk about my problems with my friends^*^				0.72
I have friends who are willing to comfort and share with me^*^				0.80
There are people who always take care of my feelings^*^				0.58
Floor effect	3.36	0.62	2.85	3.97
Ceiling effect	4.03	5.59	10.57	13.48
Cronbach's alpha	0.84	0.79	0.89	0.76
Mean (SD)	1.50 (0.80)	1.94 (0.64)	1.26 (0.81)	1.19 (0.80)

* *Reversed score*.

The floor and ceiling effects were low at <15%, while the internal consistency reliabilities were high in all four factors, with the Cronbach's alpha coefficients for the finance, study, family, and friend factors being 0.84, 0.79, 0.89, and 0.76, respectively.

### Correlation Between Severe SoMD and Four Components of Scale FSFF

**Figure 2:**
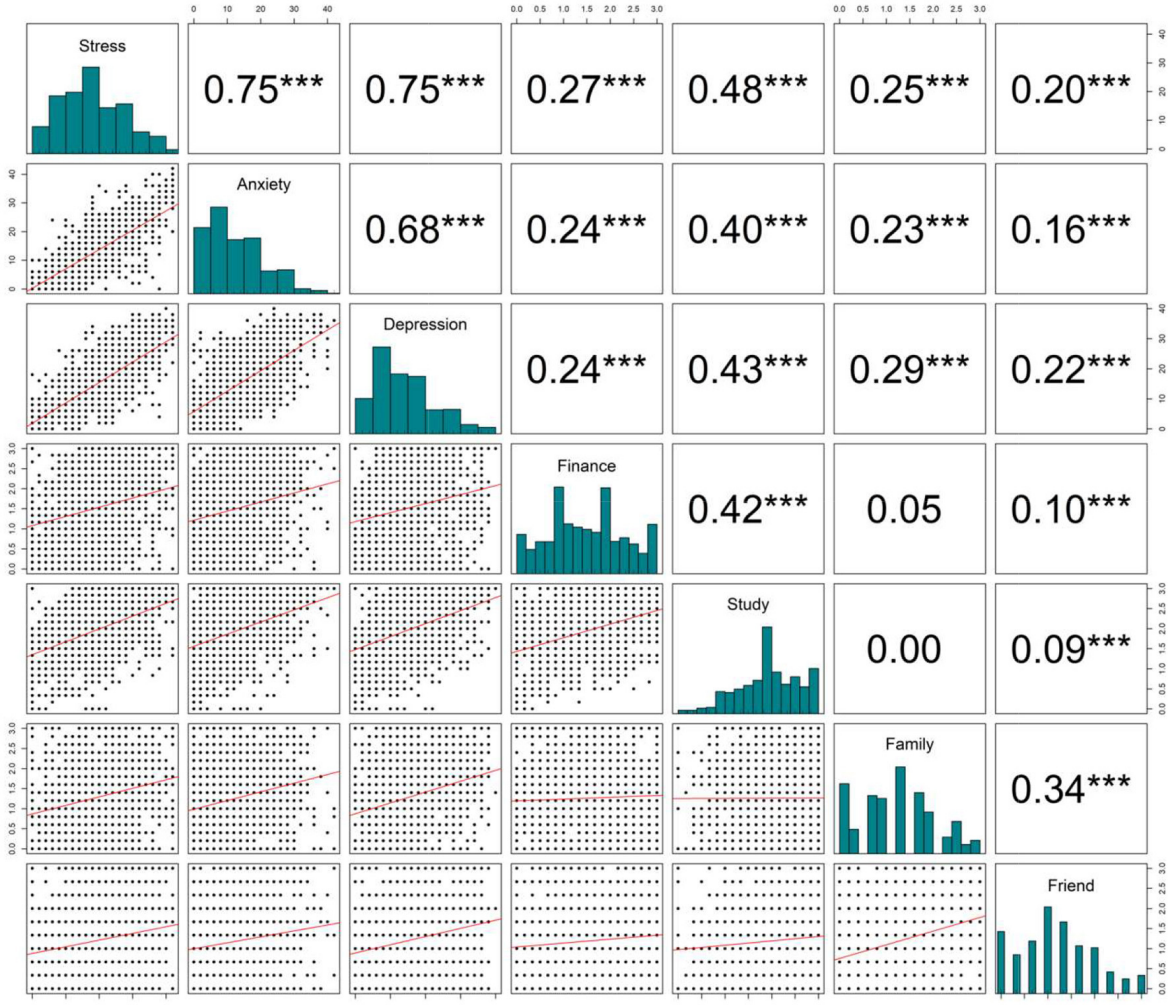
Correlation matrix of DASS21 score (depression, anxiety, and stress scores) and four components of FSFF scale. ***p < 0.001.

Figure 2 shows the correlation between the DASS-21 score (stress, anxiety, depression) and four components (finance, study, family, friend) of scale FSFF. The stress, anxiety, and depression scores were strongly correlated, with correlation coefficients ranging from 0.68 to 0.75, while the correlation among four components of the FSFF scale was weak to moderate, with coefficients ranging from 0 to 0.43. Regarding the relation between DASS-21 subscales and FSFF subscales, the strongest relationships were found between the study aspect of the FSFF scale and DASS-21 subscales, with correlation coefficients ranging from 0.40 to 0.48, *p* < 0.001.

### Relationship Between Severe SoMD and Four Components of the FSFF Scale

Table 3 shows the logistic regression of the relationship between severe mental health problems and four components of the FSFF scale. The results from fully adjusted models were consistent with those from the partially adjusted models. Conditional on students' demographic characteristics, all four aspects of the FSFF scale were related to the mental health problems of students. In worrying about finance, family, and friend, particularly, were associated with higher odds of having severe stress, with the OR ranging from 1.29 (95%CI: 1.11–1.51) to 1.76 (95%CI: 1.51–2.05), and 1.44 (95%CI: 1.24–1.67), respectively. Students who felt worried about their studies were 3.72 times (95%CI: 3.01–4.59) more likely to experience severe stress.

**Table 3:** Relationship between FSFF scale and severe symptoms of mental disorders development.

	**Partially adjusted model**			**Fully adjusted model**		
	**OR**	**95 CI**	***P*-value**	**OR**	**95 CI**	***P*-value**
**Severe stress**						
Finance	1.23	[1.06; 1.42]	0.005	1.29	[1.11; 1.51]	0.001
Study	3.76	[3.08; 4.60]	0.000	3.72	[3.01; 4.59]	0.000
Family	1.85	[1.60; 2.15]	0.000	1.76	[1.51; 2.05]	0.000
Friend	1.40	[1.20; 1.62]	0.000	1.44	[1.24; 1.67]	0.000
**Severe anxiety**						
Finance	1.18	[1.02; 1.37]	0.026	1.23	[1.05; 1.43]	0.008
Study	2.87	[2.36; 3.48]	0.000	2.84	[2.32; 3.48]	0.000
Family	1.68	[1.44; 1.95]	0.000	1.63	[1.40; 1.90]	0.000
Friend	1.16	[1.00; 1.34]	0.049	1.18	[1.01; 1.37]	0.029
**Severe depression**						
Finance	1.36	[1.16; 1.59]	0.000	1.37	[1.17; 1.61]	0.000
Study	3.22	[2.62; 3.94]	0.000	3.11	[2.51; 3.84]	0.000
Family	1.79	[1.53; 2.09]	0.000	1.75	[1.49; 2.05]	0.000
Friend	1.46	[1.25; 1.72]	0.000	1.46	[1.24; 1.72]	0.000

*All models are adjusted for age, gender, monthly living expenses, GPA, family medical history*.

Similar patterns were found in students who experienced severe anxiety and severe depression. Among these, students worrying about finance, study, family, and friends were likely to have severe anxiety, with OR ranging from 1.18 to 2.84; and had a higher likelihood of having severe depression, with OR ranging from 1.37 to 3.11. All results were significant, with *p* < 0.001.

## Discussion

In this study, we found a high prevalence of severe SoMD among foreign language students. Specifically, the stress rates of students in the research sample were severe (8.4%), and extremely severe (7.9%). The anxiety rate of students in the study sample was severe (12.4%); approximately one-fourth of participants were at an extremely severe level (22.6%). The rate of depression of students in the study sample was severe (11.1%), and extremely severe (10%), respectively. This rate was higher compared to previous studies (24, 25).

Regarding gender characteristics and the level of severe SoMD among college students, another study of high school students also showed a high percentage of students suffering from depression, anxiety, and stress, with depression and anxiety having a statistically significant association with gender (26). Females had higher levels of both depression and anxiety and this difference was statistically significant. Other studies have also found that gender differences and depression and anxiety among students were significantly related, and depression tended to be less prevalent among males than females (27, 28). The differences between male and female students in depression and anxiety prevalence could be explained by the average personality traits of both genders. Females generally have higher levels of neuroticism than males (29). Therefore, this research may be impacted by the inclusion of a higher number of female than male participants. This gender imbalance is a characteristic of language schools in Vietnam.

In this study, the percentage of students with depression, anxiety, and stress was positively correlated with a number of factors, such as financial situation, family, friends, and academic problems. In particular, anxiety about academic problems can cause students to experience higher stress levels than other problems. A correlation between demographic factors (such as economic conditions and family support) and depression and anxiety symptoms was found in the study by Abdel Wahed and Hassan (30). They found that higher depression scores were associated with increasing age, low socioeconomic standards, and geographical location. Socioeconomic status, especially the financial status of the family, is a factor that affects the quality of life and mental health of students (31–33). Besides economic factors, social support from family and friends is correlated with better mental health among students. These findings are similar to the research results of authors such as Chernomas and Shapiro (34), Usher and Curran (35), and Hefner and Eisenberg (36).

Regarding the correlation between learning issues and mental health problems, especially stress levels, our research shows that anxiety about studying can cause almost four times more stress than other influencing factors. A similar result was also found in the study conducted by Mofatteh (37). The author asserts that numerous college-related academic stressors can lead to S.A.D (Stress, Anxiety, Depression) in students. One of the factors that was strongly present in many of the studies evaluated in this review was degree-related issues and the pressure to graduate. The relationship between study pressure and stress and anxiety levels in foreign language students was shown to be similar in studies by Köksal et al. (38); Marcos-Llinás and Garau (39).

Thus, it can be said that the four factors affecting students' severe SoMD (stress, anxiety, and depression) that were investigated in this study (namely, financial problems, familial support, friends, and academic problems) were statistically significant and similar to previous studies. In the scope of this study, studying was the factor that had the most significant impact on students' mental health problems in all three aspects: stress, depression, and anxiety. Next are family, friends, and finally financial issues.

These are remarkable findings and could greatly assist schools and psychologists in developing ways to reduce levels of stress, depression, and anxiety, and thereby improve the quality of students' mental health. In fact, in response to the findings of this study, several online workshops were held as part of UPC activities for students in several universities to teach students about mental health, how to balance their emotions, and the importance of healthy activities.

## Limitations

The findings of this study should be interpreted in the context of potential limitations. Firstly, as this is a cross-sectional study, students' psychological problems could only be measured for a short period of time. Further research needs to be conducted over an extended period to determine whether the manifestations of depression, anxiety, and stress are long-term problems for foreign language students. Secondly, given that many previous studies have shown that a higher percentage of female students suffer from depression, the greater number of female than male students included in this study may have resulted in more students reporting severe SoMD than would have if the genders were balanced; the results in this regard would be more accurate if the proportion of male participants is higher. Thirdly, using DASS-21 only helped us to capture the severity of symptoms of MHD in students, not a diagnosis for mental disorders. Fourthly, the participants in this study comprised only 35.6% of the total number of students at the University of Languages and International Studies, which might introduce selection bias.

## Conclusion

The research showed that students of the University of Foreign Languages in Hanoi, Vietnam have a high prevalence of severe SoMD. Studying and family are two factors that significantly impacted the levels of stress, anxiety, and depression of students, and students who had close connections and received care and support from their families were shown to have better mental health. These findings also suggest that teachers and universities should pay special attention to female students because they tend to be more prone to mental disorders than male students. Schools and educators should promote information about the role of families in students' SoMD and develop appropriate learning strategies to ensure both students' mental health and their learning quality.

## Data Availability Statement

The raw data supporting the conclusions of this article will be made available by the authors, without undue reservation.

## Ethics Statement

The study was approved by the Institutional Review Board of Hanoi University of Public Health in Vietnam (IRB decision no 325/2021/YTCC-HD3). The patients/participants provided their written informed consent to participate in this study.

## Author Contributions

DL and NT conceived of the study. NT and KL performed the official statistical analyses and interpreted the results. NT, DL, and NP wrote the manuscript. TA, NG, NL, and DN provided the critical revision of the manuscript for important intellectual content. All authors read and approved the final manuscript.

## Funding

This research was funded by the University of Languages and International Studies, Vietnam National University, Hanoi under grant number N.21.01.

## Conflict of Interest

The authors declare that the research was conducted in the absence of any commercial or financial relationships that could be construed as a potential conflict of interest.

## Publisher's Note

All claims expressed in this article are solely those of the authors and do not necessarily represent those of their affiliated organizations, or those of the publisher, the editors and the reviewers. Any product that may be evaluated in this article, or claim that may be made by its manufacturer, is not guaranteed or endorsed by the publisher.
