# Supplementary material for: Severe Symptoms of Mental Disorders Among Students Majoring in Foreign Languages in Vietnam: A Cross-Sectional Study
Source: Front Public Health. 2022 May 30;10:855607. doi: 10.3389/fpubh.2022.855607 (PMC9196266; doi:10.3389/fpubh.2022.855607)
Supplement: Supplementary file 1 [file Table_1.DOCX]

Supplemental table 1: Bilingual version of the FSFF scale and scoring guide.

|  |  | Hoàn toàn không đồng ý  Totally disagree | Không đồng ý  Disagree | Tương đối đồng ý  Agree | Hoàn toàn đồng ý  Totally agree | Subscale |
| --- | --- | --- | --- | --- | --- | --- |
|  |  | 0 | 1 | 2 | 3 |  |
| Item 1 | Tôi lo nghĩ về nguồn kinh phí đóng học  I’m worried about the cost of tuition |  |  |  |  | Finance |
| Item 2 | Tôi lo nghĩ về các chi phí sinh hoạt hằng ngày  I’ve calculated daily living expenses |  |  |  |  | Finance |
| Item 3 | Tôi lo tìm việc làm thêm hàng tháng  I worry about finding a part-time job every month |  |  |  |  | Finance |
| Item 4 | Hỗ trợ kinh phí từ gia đình không đủ chi phí sinh hoạt và học tập cho tôi  My family’s financial support is not enough for me to live and study |  |  |  |  | Finance |
| Item 5 | Tôi mong được hỗ trợ kinh phí học tập và sinh hoạt từ nguồn ngoài gia đình  I hope to receive financial support from sources outside my family |  |  |  |  | Finance |
| Item 6 | Tôi lo lắng tới kinh phí học tập và sinh hoạt hằng ngày của mình  I worry about tuition and daily living expenses |  |  |  |  | Finance |
| Item 7 | Tôi phân vân về nghề nghiệp trong tương lai  I wonder about my future career |  |  |  |  | Study |
| Item 8 | Tôi gặp những khó khăn trong giải quyết các nhiệm vụ học tập  I have difficulty solving academic tasks |  |  |  |  | Study |
| Item 9 | Tôi cố tìm ra phương pháp học tập hiệu quả cho cá nhân  I try to find a way to study that works for me personally |  |  |  |  | Study |
| Item 10 | Tôi bị quá tải với các deadline học tập các môn học  I am overloaded with academic deadlines |  |  |  |  | Study |
| Item 11 | Tôi ám ảnh về thành tích học tập của cá nhân  I’m obsessed with personal academic achievement |  |  |  |  | Study |
| Item 12 | Tôi cần sự hỗ trợ trong quá trình học tập  I need help with my studies |  |  |  |  | Study |
| Item 13* | Tôi có thể nói những vấn đề của mình với gia đình  I can talk about my problems with my family |  |  |  |  | Family |
| Item 14* | Tôi nhận được sự chia sẻ và động viên từ gia đình  I received support and encouragement from my family |  |  |  |  | Family |
| Item 15* | Tôi thấy thoải mái mỗi khi nói chuyện với các thành viên trong gia đình  I feel comfortable talking to my family members |  |  |  |  | Family |
| Item 16* | Gia đình hỗ trợ tôi đưa ra quyết định  My family supports me in making decisions |  |  |  |  | Family |
| Item 17* | Gia đình thực sự cố gắng giúp tôi  My family really tries to help me |  |  |  |  | Family |
| Item 18* | Tôi có thể nói những vấn đề của mình với bạn bè  I can talk about my problems with my friends |  |  |  |  | Friend |
| Item 19* | Tôi có những người bạn sẵn sàng an ủi và chia sẻ cùng tôi  I have friends who are willing to comfort and share with me |  |  |  |  | Friend |
| Item 20* | Có người đặc biệt quan tâm tới cảm xúc của tôi  There are people who always take care of my feelings |  |  |  |  | Friend |

*The score of these item need to be reversed
